# Enabling Thin-Edged Part Machining of Nomex Honeycomb Composites via Optimizing Variable Angle of Disc Cutters

**DOI:** 10.3390/ma16165611

**Published:** 2023-08-13

**Authors:** Xinman Yuan, Kexin Zhang, Huiting Zha, Jie Xu, Ge Song, Wenjun Cao, Pingfa Feng, Feng Feng

**Affiliations:** 1 AVIC Chengdu Aircraft Industrial (Group) Co., Ltd., Chengdu 610073, China; yz880417@163.com (X.Y.); rommel_sg@163.com (G.S.); 13678026768@163.com (W.C.); 2 Division of Advanced Manufacturing, Shenzhen International Graduate School, Tsinghua University, Shenzhen 518055, China; zhang-kx21@mails.tsinghua.edu.cn (K.Z.); xu-j22@mails.tsinghua.edu.cn (J.X.); feng.pingfa@sz.tsinghua.edu.cn (P.F.); 3 School of Mechanical and Automotive Engineering, Xiamen University of Technology, Xiamen 361024, China

**Keywords:** ultrasonic vibration machining, variable angle of disc cutter, Nomex honeycomb composites

## Abstract

Machining Nomex honeycomb composites (NHCs), which are widely-used materials in the aerospace industry, is an imperative process to obtain desired profiles. However, when machining NHCs to obtain a thin-edged surface, some problems can arise due to large cutting forces. To avoid these defects, a method of ultrasonic vibration machining with variable angles of the down milling disc cutter was proposed in this study. The processing principles and motion characteristics of this method were elaborated. A theoretical model of its cutting process was established. The principle of cutting force reduction was qualitatively analyzed based on the model, and an experimental validation was conducted. The results demonstrated that, due to a smaller swing angle in each pass, the proposed method could reduce the fractal dimension of the machined surface by 6.01% compared to 1° with 10° of angle in each pass. And severe machining defects were decreased. Additionally, comparing the process of the fixed 10° angle of ultrasonic vibration machining with the process of a 1° angle in a pass, cutting force can be significantly reduced by 33.5%, demonstrating the effectiveness of the proposed method which improved surface quality by reducing cutting forces.

## 1. Introduction

Lightweight and excellent performance under extreme conditions are primary requirements in the aerospace manufacturing industry [1]. Nomex honeycomb composites (NHCs) fulfill these requirements by offering high specific strength and stiffness [2,3], enabling weight reduction while maintaining sufficient strength and stiffness. In terms of physicochemical properties, NHCs exhibit good corrosion resistance, flame retardancy, and dielectric properties [4,5], which enhance their performance under extreme conditions. Moreover, the honeycomb structure of NHCs is inspired by the natural honeycomb, featuring a periodic topology that provides excellent impact resistance [6], negative Poisson’s ratio [7], negative thermal expansion [8], compressive torsion [9], and negative stiffness [10]. Consequently, NHCs have extensive applications in aerospace, transportation, construction, and other industries [11].

The widespread use of NHCs has created an urgent need for their efficient machining processes. Traditional machining methods for NHCs include milling [12] and thin-blade tool cutting [13,14]. However, these methods suffer from low efficiency, severe machining defects, large cutting forces, rapid tool wear, and severe dust pollution during the process [15,16,17,18]. To address these problems, researchers have proposed many improvements to the machining processes for NHCs. Xie et al. [19] used a combined milling tool to avoid dust generation during machining. In addition, because process parameters are of high importance, some researchers pay attention to the process parameters. As for the entry direction of the tool, both Xu et al. and Jiang et al. [20,21] proposed a method based on changing the entry direction of the disc cutter to suppress damage during machining. These methods achieved a certain level of enhancement in the machining qualities of NHCs but are not suitable for conditions with large cutting forces.

In comparison to traditional machining, ultrasonic vibration machining can effectively reduce cutting forces because of its intermittent cutting [22,23]. And many researchers have verified the effectiveness of ultrasonic vibration machining by experimental and theoretical methods. Ahmad et al. [24] compared the cutting forces of disc cutters on NHCs under ultrasonic and traditional machining conditions and found that ultrasonic vibration machining significantly reduced cutting forces. Furthermore, ultrasonic machining greatly improves the surface qualities of NHCs. Kang et al. [25] compared the machined surfaces of NHCs under ultrasonic and traditional machining, respectively, and the results showed that the machined surface under ultrasonic machining conditions can greatly suppress the generation of defects. Xiang et al. [26] used a disc cutter under a longitudinal-torsional vibration condition to machine NHCs and found that the superimposition of torsional vibration on longitudinal vibration not only increased the instantaneous cutting speed of the disc cutter but also facilitated material fracture, resulting in shorter burrs, lower burr rates, and fewer tearing defects. In terms of force model during the milling process, Wojciechowski et al. [27] proposed a comprehensive micro milling force model which had a high accuracy of predicted forces in cutting. Yuan et al. [28] developed an accurate mechanistic cutting force model for micro end-milling and the results showed that the predicted and the experimental cutting forces had similar variation patterns and closely matched amplitude levels. Xiang et al. [29] proposed a cutting force model and verified the reduction of cutting force using an ultrasonic vibration machining method to cut the NHCs.

However, when machining NHCs with complex profiles and ultra-thin edges, defects such as tearing, deformation, uneven cutting profiles, cracks, and cell detachment from the base can occur even under the ultrasonic vibration machining condition [30,31]. Hence, some researchers have proposed different methods related to the fixing method, path strategy of the cutting tool, and new structural tools to reduce machining errors on complex surfaces and improve surface quality. In terms of the fixing method, Jin [32] proposed a new fixing method and applied it to the milling experiment. By analyzing the force in the milling process, the effectiveness of the proposed fixing method based on the magnetic field and the friction principle was verified. As for the path strategy of the cutting tool, Sha et al. [33] modeled and compensated for geometric errors in the machining positions of NHCs on complex surfaces to enhance machining accuracy. As for the new tool, Sun et al. [34] proposed a method of semi-circular arc tool ultrasonic plunge cutting for NHCs, which significantly improved the surface qualities of machined surfaces by combining ultrasonic vibration with plunge cutting. Nevertheless, few methods contribute to both force reduction and surface quality improvement by ameliorating the machining process with ultrasonic vibration condition.

Considering the conclusion from Xiang’s [29] study which showed that with the increase of the deflected angle of the thin-blade tool, both cutting forces with and without the ultrasonic machining condition increase, the swing angle of the disc cutter is regarded as an important factor during machining. To enhance the machining qualities of NHCs and reduce defects such as tearing and deformation during machining, this paper innovatively proposes a machining method that combines the variable angle of the disc cutter technique with ultrasonic vibration machining for NHCs. The effectiveness of this method in reducing cutting forces was verified with a theoretical mechanical model. Additionally, experiments were conducted to investigate the influence of the proposed machining method on cutting forces during the cutting process and improvement in the surface qualities of NHCs.

## 2. Analysis on Ultrasonic Vibration Machining Process

### 2.1. Motion Analysis

**Figure 1:**
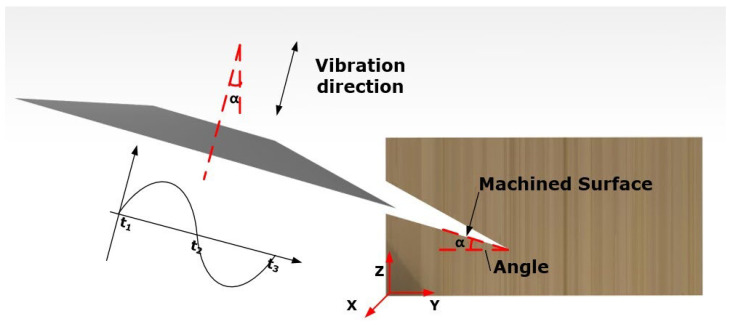
Schematic diagram of ultrasonic vibration machining with the fixed angle of disc cutter.

Generally, to machine the desired profiles of NHCs, the disc cutter cuts the NHCs by applying ultrasonic vibration machining with a fixed angle. The schematic diagram of this process can be shown in Figure 1. According to the angle α of the machined surface, the axis of the disc cutter swings at a fixed angle α relative to the Z direction. Additionally, ultrasonic vibration is applied to the axial of the disc cutter.

Based on the understanding of ultrasonic vibration machining, the displacement, velocity, and acceleration of the disc cutter under ultrasonic vibration conditions can be expressed by Equations (1)–(3) [29].

(1) $$S_{u}=Asin(2\pi ft)$$

(2) $$v_{u}=2\pi fAcos(2\pi ft)$$

(3) $$a_{u}=-4{\left(\pi f\right)}^{2}Asin(2\pi ft)$$

where $S_{u}$, $v_{u}$ and $a_{u}$ respectively represent the displacement, velocity, and acceleration of the disc cutter under axial ultrasonic vibration conditions. A is the ultrasonic amplitude. f is the ultrasonic frequency.

**Figure 2:**
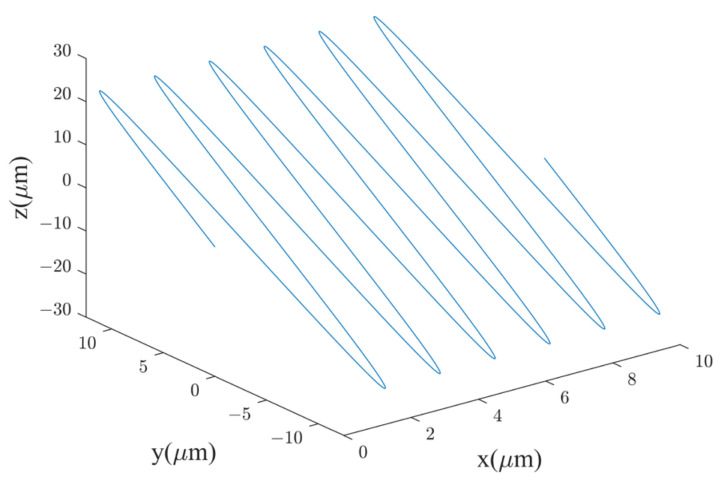
Motion trajectory of the disc cutter under a fixed angle during ultrasonic vibration machining.

After decomposing the equation of ultrasonic vibration displacement into X, Y, and Z directions, the motion trajectory of the disc cutter can be obtained as shown in Equations (4)–(6). Additionally, the velocity and acceleration of the disc cutter in the X, Y, and Z direction are given by Equations (7)–(12) [29]. Figure 2 illustrates the motion trajectory of the disc cutter under a fixed angle during ultrasonic vibration machining.

(4) $$S_{x}=v_{f}t$$

(5) $$S_{y}=Asin\alpha sin(2\pi ft)$$

(6) $$S_{z}=Acos\alpha sin(2\pi ft)$$

(7) $$v_{x}=v_{f}$$

(8) $$v_{y}=2\pi fAsin\alpha cos(2\pi ft)$$

(9) $$v_{z}=2\pi fAcos\alpha cos(2\pi ft)$$

(10) $$a_{x}=0$$

(11) $$a_{y}=-{\left(2\pi f\right)}^{2}Asin\alpha sin(2\pi ft)$$

(12) $$a_{z}=-{\left(2\pi f\right)}^{2}Acos\alpha sin(2\pi ft)$$

where $v_{f}$ is the feed speed of the tool. α is the angle between the axis of the tool and the Z direction.

**Figure 3:**
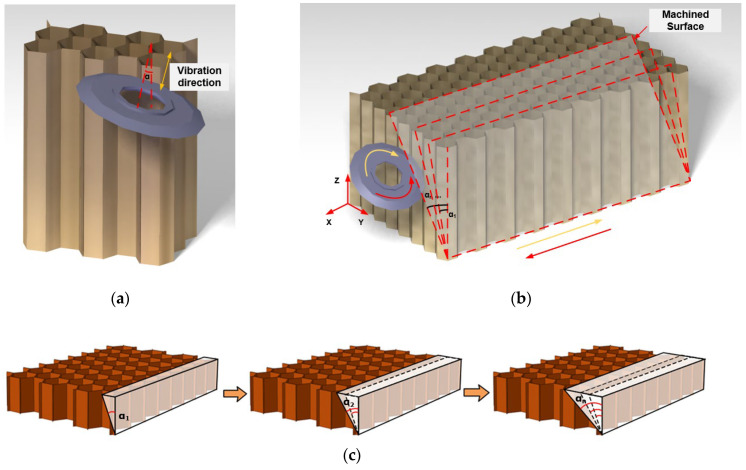
Ultrasonic vibration machining process with variable angle of the disc cutter. (**a**) Schematic diagram of the process of ultrasonic vibration machining with variable angle of the disc cutter; (**b**) Tool motion during machining; (**c**) Schematic diagram of machined surfaces during machining.

Compared with the method of a disc cutter that cuts the NHCs with a fixed angle under the ultrasonic vibration condition, the method of ultrasonic vibration machining using a variation-angle disc cutter combines ultrasonic vibration machining and the technology of variable angle of the tool. Figure 3 illustrates the schematic diagram of the process of ultrasonic vibration machining with a variable angle of the disc cutter. As shown in Figure 3a, the ultrasonic vibration is applied in the axial direction of the disc cutter and the angle that should be changed in the technology of the variable angle of the tool is the angle α of the tool between the tool axis and the Z direction. Figure 3b,c depict the specific process. Instead of milling the NHCs at a fixed angle corresponding to the desired machined surface, the disc cutter gradually varies its angle from a small angle $\alpha_{1}$ to a large angle $\alpha_{n}$ to machine the corresponding surfaces until the final machined surface is obtained. The disc cutter adopts a down milling approach for machining the NHCs, where the disc cutter rotates clockwise when the feed direction is the same as the negative direction of the X axis. Conversely, when the feed direction is the same as the positive direction of the X axis, the disc cutter rotates counterclockwise.

### 2.2. Cutting Force Modeling

This study compares the machining conditions of the disc cutter in four states when cutting NHCs: (1) traditional ultrasonic machining, (2) ultrasonic vibration machining with variable angle of the disc cutter, (3) ultrasonic vibration machining with variable angle of the up milling disc cutter, and (4) ultrasonic vibration machining with variable angle of the down milling disc cutter.

#### 2.2.1. Traditional Ultrasonic Machining

**Figure 4:**
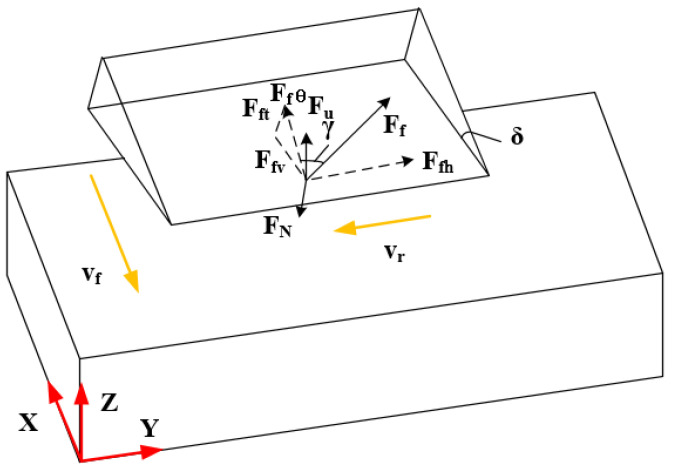
Force model of traditional ultrasonic machining.

It can be observed that, when the disc cutter feeds in the negative direction of the X axis shown in Figure 3b, the disc cutter rotates clockwise. Thus, considering the instantaneous condition of the contact point between the tool and the workpiece, Figure 4 illustrates the force model in traditional ultrasonic machining when cutting NHCs.

The average impact force of the disc cutter due to ultrasonic vibration in a single period is Equation (13) [25].

(13) $$F_{u}^{\prime }=\frac{t_{s}}{T}m\bar{a_{u}}=2\pi f^{2}Am(\cos\left(2\pi ft_{2}\right)-\cos(2\pi ft_{1}))$$

where $t_{s}$ is the effective time of ultrasonic cutting of NHCs by the disc cutter in one cycle. $t_{2}$ is the moment when the disc cutter and the NHCs begin to separate. $t_{1}$ is the moment when the disc cutter starts to contact the NHCs. T is an ultrasonic vibration period. And m is the mass of the disc cutter.

In a vibration period, $v_{f}$ and $v_{r}$ are the feed speed and linear velocity of the tool, respectively. The pressure $F_{N}$ of the front cutter face on the front cutter face is caused by ultrasonic vibration, and then the average pressure $F_{uN}=\frac{t_{s}}{T}F_{N}$ and friction force $F_{f}=\mu F_{uN}$ is calculated. Specifically, $F_{fh}$ and $F_{f\theta }$ are the components of the friction force $F_{f}$ on the front surface of the tool. And $F_{ft}$ and $F_{v}$ are the components of $F_{f\theta }$ on the cross-section of the tool. Moreover, γ is the fixed angle between the friction force $F_{f}$ and $F_{f\theta }$; that is, $\gamma =\arctan\left(\frac{F_{fh}}{F_{f\theta }}\right)=\arctan(\frac{v_{r}}{v_{f}}\cos\delta )$. The total forces $F_{x}$ and $F_{z}$ in the X direction and the Z direction are shown in Equations (14) and (15) [25].

(14) $$F_{x}=F_{uN}(\mu \cos\gamma \cos\delta +\sin\delta )$$

(15) $$F_{z}=F_{uN}(\mu \cos\gamma \sin\delta -\cos\delta )+F_{u}^{\prime }$$

where μ is the friction coefficient and δ is the wedge angle of the disc cutter.

If the ultrasonic vibration is not applied on the disc cutter, the force of the tool $F_{xt}$ and $F_{zt}$ in the feed direction and the axial direction are shown in Equations (16) and (17) [25]. Because of $F_{N}>F_{uN}$ and $F_{u}^{\prime }<0$, the ultrasonic vibration machining of NHCs is beneficial to reduce the cutting force.

(16) $$F_{xt}=F_{N}(\mu \cos\gamma \cos\delta +\sin\delta )$$

(17) $$F_{zt}=F_{N}(\mu \cos\gamma \sin\delta -\cos\delta )$$

#### 2.2.2. Variable Angle of Disc Cutter

**Figure 5:**
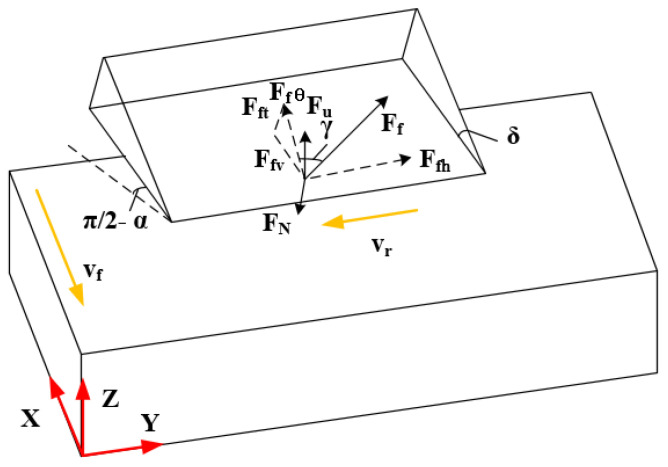
Force model of ultrasonic vibration machining with variable angle of the disc cutter.

Figure 5 shows the analysis of the forces in the variable angle of the disc cutter, which is based on traditional ultrasonic machining (Figure 4). Similarly, when the disc cutter cuts the material, the contact point between the tool and the workpiece is considered. When the disc cutter feeds in the negative direction of the X axis shown in Figure 3b, the disc cutter rotates clockwise. The forces $F_{xv}$ and $F_{zv}$ in the X and Z directions are given by Equations (18) and (19) [25].

(18) $$F_{xv}=F_{uN}(\mu \cos\gamma_{v}\cos\left(\frac{\pi }{2}-\alpha +\delta \right)+\sin\left(\frac{\pi }{2}-\alpha +\delta \right))$$

(19) $$F_{zv}=F_{uN}(\mu \cos\gamma_{v}\sin\left(\frac{\pi }{2}-\alpha +\delta \right)-\cos\left(\frac{\pi }{2}-\alpha +\delta \right))+F_{u}^{\prime }$$

where α is the swing angle of the disc cutter and it gradually increases with each disc cutter moving process. $\gamma_{v}$ is the angle between the friction force $F_{F}$ and its component force $F_{F\theta }$; that is, $\gamma_{v}=\arctan\left(\frac{F_{Fh}}{F_{F\theta }}\right)=\arctan(\frac{v_{r}}{v_{F}}\cos\left(\frac{\pi }{2}-\alpha +\delta \right))$.

It can be seen that, if a larger angle is used, a larger angle will cause the increase of the force $F_{xv}$ and $F_{zv}$. On the contrary, if the method of variable angle is used, where the smaller angle is used first, it can effectively reduce the cutting force of the disc cutter when cutting NHCs.

#### 2.2.3. Variable Angle of Up Milling Disc Cutter

**Figure 6:**
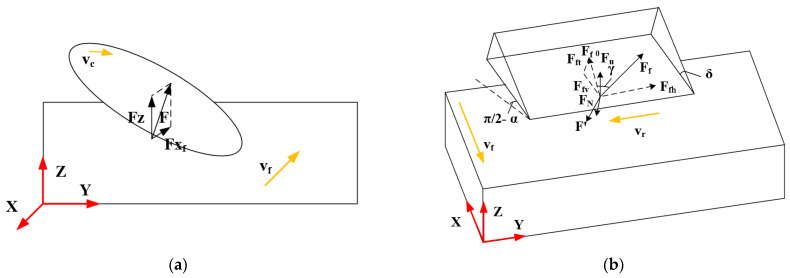
Schematic diagram of ultrasonic vibration machining with variable angle of the up milling disc cutter. (**a**) Diagram of the force of disc cutter during the machining process; (**b**) Force model of the process.

Figure 6a shows the schematic diagram of ultrasonic vibration machining with a variable angle of the up milling disc cutter. When the disc cutter is processed by up milling, as the direction of the cutting speed of the tool is opposite to the direction of the movement of the workpiece, there is upward resultant force F on the workpiece. Because the workpiece is affected by the upward force, the workpiece tends to be lifted during processing, which is likely to cause the NHCs and the bottom platform to appear the trend of debonding.

As shown in Figure 6b, the disc cutter is also subjected to the reaction force F′ of the workpiece on the tool. The force $F_{xv}$ and $F_{zv}$ of the tool in the X direction and the Z direction is as shown in Equations (20) and (21) [25].

(20) $$F_{xv}=F_{uN}(\mu \cos\gamma_{v}\cos\left(\frac{\pi }{2}-\alpha +\delta \right)+\sin\left(\frac{\pi }{2}-\alpha +\delta \right))+F_{xf}^{\prime }$$

(21) $$F_{zv}=F_{uN}(\mu \cos\gamma_{v}\sin\left(\frac{\pi }{2}-\alpha +\delta \right)-\cos\left(\frac{\pi }{2}-\alpha +\delta \right))+F_{u}^{\prime }-F_{z}^{\prime }$$

where $F_{xf}^{\prime }$ is the force in the X direction that the disc cutter is subjected to during the up milling process and $F_{z}^{\prime }$ is the force in the Z direction that the tool is subjected to during the up milling process.

It can be seen that the upward resultant force of the workpiece is an important factor that causes the debonding during the machining process. And the tool force in the X direction increases when the disc cutter cuts the NHCs under the up milling condition.

#### 2.2.4. Variable Angle of Down Milling Disc Cutter

**Figure 7:**
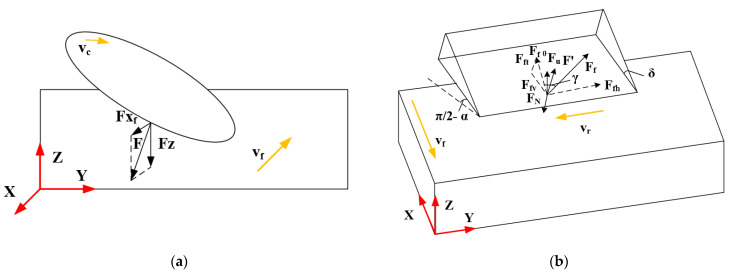
Schematic diagram of ultrasonic vibration machining with variable angle of the down milling disc cutter. (**a**) Diagram of the force of disc cutter during the machining process; (**b**) Force model of the process.

Figure 7a shows the schematic diagram of ultrasonic vibration machining with a variable angle of the down milling disc cutter. Because the disc cutter is processed in down milling, the workpiece can be subjected to the pressure $F_{N}$ and friction $F_{F}$ of the tool during the machining process. Thus, the force F of the workpiece is downward and the workpiece is subjected to downward force during the machining process, which reduces the required clamping force.

As shown in Figure 7b, the tool is also subject to the reaction force F′ of the workpiece on the tool, so the force $F_{xv}$ and $F_{zv}$ of the tool in the X direction and the Z direction is as shown in Equations (22) and (23) [25].

(22) $$F_{xv}=F_{uN}(\mu \cos\gamma_{v}\cos\left(\frac{\pi }{2}-\alpha +\delta \right)+\sin\left(\frac{\pi }{2}-\alpha +\delta \right))-F_{xf}^{\prime }$$

(23) $$F_{zv}=F_{uN}(\mu \cos\gamma_{v}\sin\left(\frac{\pi }{2}-\alpha +\delta \right)-\cos\left(\frac{\pi }{2}-\alpha +\delta \right))+F_{u}^{\prime }+F_{z}^{\prime }$$

where $F_{xf}^{\prime }$ is the force in the X direction during the down milling process and $F_{z}^{\prime }$ is the force in the Z direction during the down milling process.

It can be seen that compared with up milling, down milling is not only conducive to the reduction of the required clamping force but also beneficial to the reduction of the tool force in the X direction when the disc cutter cuts the NHCs.

## 3. Materials and Methods

### 3.1. Experimental Platform

**Figure 8:**
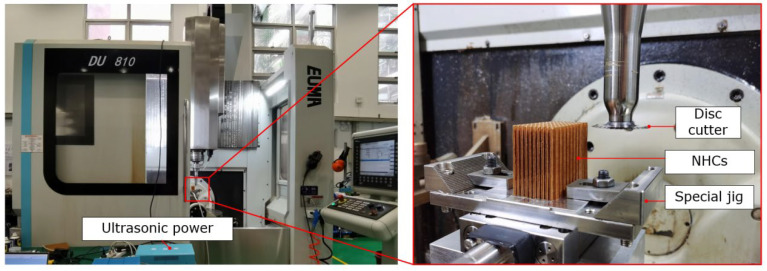
Experimental platform.

The experimental platform is shown in Figure 8. A machining center was used. The material of the workpiece was made of NHCs, which are composites combined with short aramid fiber and phenolic resin with regular hexagonal cells. And the size of the workpiece was 30 mm × 58 mm × 45 mm. A toothed disc cutter with a diameter of 50 mm was used. To generate ultrasonic vibration, an ultrasonic power (UMINT-20-1000-Y, TsingDing, Shenzhen, China) was used which could monitor the frequency. Hence, the ultrasonic machining frequency was set at 20.42 kHz and the corresponding amplitude was 27.5 μm, as measured by laser displacement sensor (KistlerLK-H025 series, Kistler, Winterthur, Switzerland). In addition, a data acquisition system (Kistler 5697A, Kistler, Winterthur, Switzerland), charge amplifier (Kistler 5080A100804, Kistler, Winterthur, Switzerland), and force sensor (Kistler 9119A, Kistler, Winterthur, Switzerland) were used to measure the cutting force.

### 3.2. Experimental Methods

The experiments are divided into two parts. The first experiment whose detailed experimental settings were illustrated in Section 3.2.1 studied the effect of variable angle on cutting force. Meanwhile, the second experiment whose exhaustive descriptions were shown in Section 3.2.2 investigated the influence of variable angle on surface quality.

#### 3.2.1. Influence of Variable Angle on Cutting Forces

To investigate the influence of variable angle and corresponding processing methods (ultrasonic machining, traditional machining, up milling, down milling) on cutting forces, four different groups were set up, with 10 different angles ranging from 1° to 10° in each group. To be specific, the disc cutter in each group cuts the NHCs with a fixed angle which can be selected from 1° to 10° under corresponding processing methods. The experimental groups 1~4 corresponded to ultrasonic up milling, ultrasonic down milling, conventional up milling, and conventional down milling, respectively. Additionally, the spindle speed, feed speed, cutting width and cutting depth are 3000 r/min, 3000 mm/min, 1 mm, and 1 mm, respectively [29], and those parameters are the same in the four groups.

**Figure 9:**
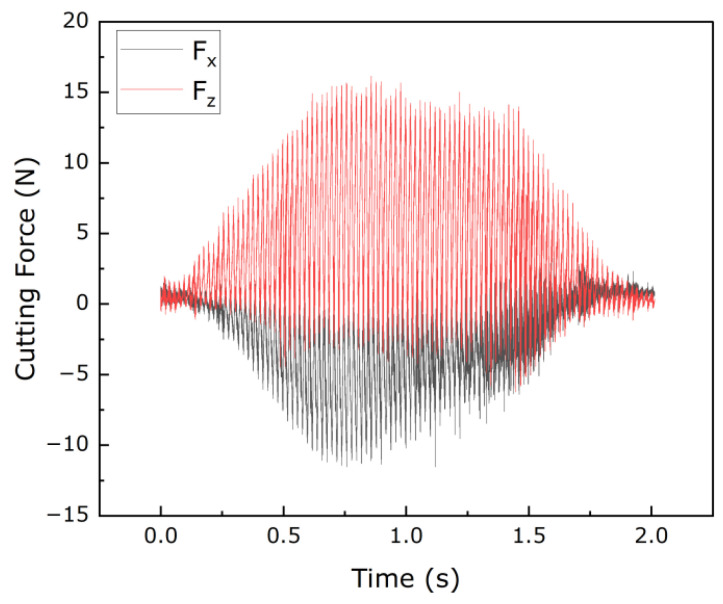
Cutting forces in machining.

According to the data on the cutting force, as shown in Figure 9, the average values of the measured cutting forces in the X and Z directions were calculated for different processing conditions.

#### 3.2.2. Influence of Variable Angle on Surface Quality

The experiment was divided into two groups, each with three repetitions. For group 5, the workpiece was processed under a fixed swing angle of 10° in ultrasonic down milling. For group 6, the workpiece was processed using the method of ultrasonic vibration machining with a variable angle of the down milling disc cutter and an angle of 5° for each pass, which means that the machined surface can be obtained by two cuts. Other values of processing parameters including spindle speed, feed speed, cutting width, and cutting depth are also consistent with the value of those parameters used in group 1 to group 4.

**Figure 10:**
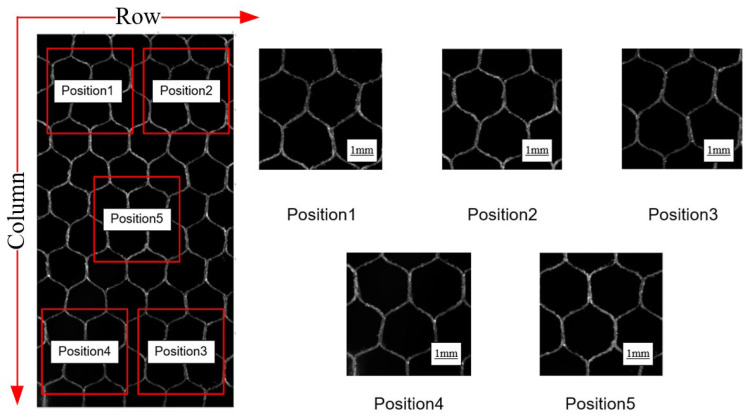
Five-point random sampling method.

To investigate the improvement of the surface qualities of NHCs by variable angle in the process of ultrasonic vibration machining, qualitative observations of the machined surfaces were made using a structured light camera and an optical microscope. Because of the porous and thin-walled characteristics of NHCs, it is difficult to observe the discontinuous machined surface. The fractal dimension, which is a core parameter representing the irregularity and fragmentary properties of a self-affine surface [35,36] can be an effective indicator to quantitatively evaluate the machined surfaces of NHCs. Therefore, the surface qualities of the machined NHCs were quantitatively characterized using fractal dimension analysis. For the calculation of the fractal dimensions of the surfaces of NHCs, a five-point random sampling method, as shown in Figure 10, was employed. In this method, after obtaining the data of the machined surfaces of NHCs, five regions with sizes of 1024 pixels × 1024 pixels were selected from the surfaces of NHCs. The positions of the center of the No 1 to No 4 regions were chosen according to the number of rows and columns. And the center of the No 5 region is the intersection of the cross-links of the centers of the other four positions. To be specific, position 1 was in row 3 and column 2, position 2 was in row 8 and column 2, position 3 was in row 8 and column 10, position 4 was in row 3 and column 10, and position 5 was in row 5 and column 6.

**Figure 11:**
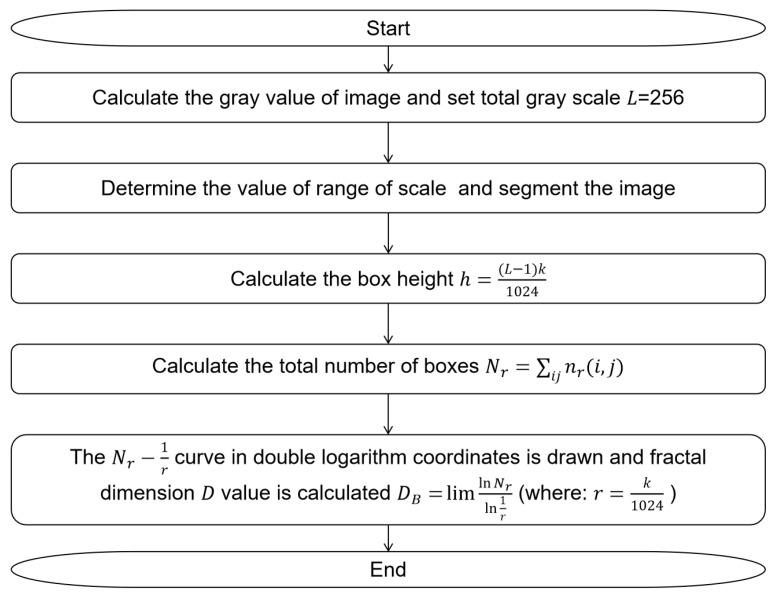
The flow chart of the box-counting algorithm.

Then, the box-counting algorithm, which is a commonly used algorithm, was used to calculate the fractal dimension of the selected region. The box-counting algorithm is measured by counting the minimum number of boxes that cover the image surface [36]. And the flow chart of the box-counting algorithm is shown in Figure 11.

After obtaining the values of the fractal dimensions of five positions, the final fractal dimension of the machined surface was the mean value of the fractal dimensions of five positions.

## 4. Results

### 4.1. Analysis of Cutting Forces for Different Angles

**Figure 12:**
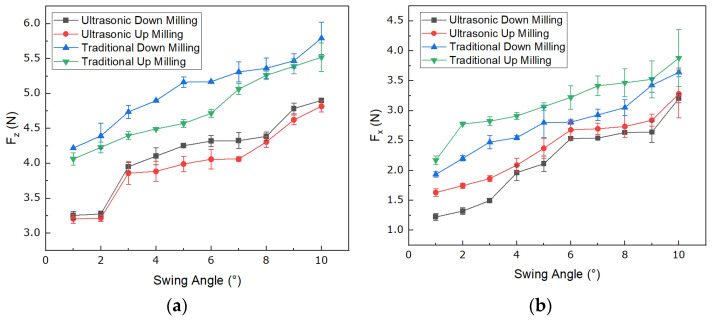
Analysis of cutting forces for different angles. (**a**) Cutting forces in the Z direction; (**b**) Cutting forces in the X direction.

The measured cutting forces of NHCs under different processing conditions are shown in Figure 12. The influence of ultrasonic vibration machining on cutting forces can be observed in Figure 12, indicating that ultrasonic vibration machining can effectively reduce cutting forces compared to conventional machining.

Regarding the effect of variable angle on cutting forces, Figure 12a shows that, as the angle decreases, the cutting forces in the Z direction decrease by 33.5% for ultrasonic down milling, 33.6% for ultrasonic up milling, 27.1% for conventional down milling, and 26.4% for conventional up milling. Additionally, Figure 12b shows that, as the angle decreases, the cutting forces in the X direction decrease by 62.0% for ultrasonic down milling, 50.3% for ultrasonic up milling, 47.0% for conventional down milling, and 44.1% for conventional up milling. The results of the reduction in the cutting force with decreased angles under four diverse processing methods are shown in Table 1.

Regarding the difference between up milling and down milling in terms of cutting forces, Figure 12b shows that the average cutting force of all angles in ultrasonic down milling is 9.44% lower than that of ultrasonic up milling, and the average cutting force in conventional down milling is 11.01% lower than that of conventional up milling. Therefore, down milling can effectively reduce the cutting forces in the X direction.

Based on the above results, it can be concluded that ultrasonic vibration machining with a variable angle of the down milling disc cutter can effectively reduce cutting forces. In Equations (18)–(23), it was clearly illustrated that the swing angle is one of the most important factors that influence the value of cutting force. Specifically, if a larger angle is used, a larger angle will cause the increase of the force $F_{xv}$ and $F_{zv}$. On the contrary, if the method of variable angle is used, that is, the smaller angle is used first, it can effectively reduce the cutting force of the disc cutter when cutting NHCs. Combining the results of the experiments and theoretical analysis, it was shown that these experimental results were consistent with the trend of the relationship between the angle and force obtained in Equations (18)–(23), indicating that the method of ultrasonic vibration machining with variable angle of the disc cutter which firstly applies a smaller angle of the tool can effectively reduce cutting forces compared to directly using a larger fixed tool angle.

### 4.2. Qualitative Analysis of Machined Surfaces of NHCs

**Figure 13:**
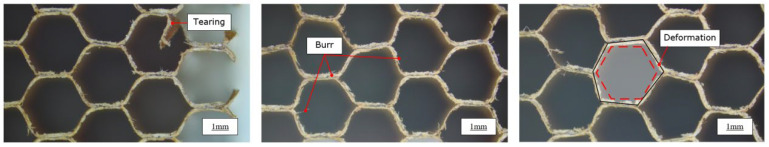
The surface of the workpiece is machined without ultrasonic vibration machining with variable angle of the disc cutter.

**Figure 14:**
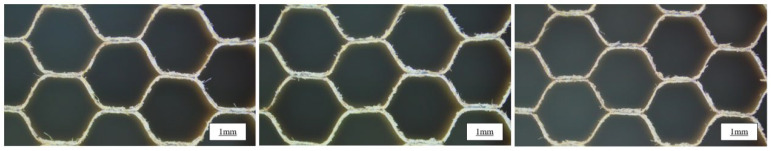
The surface of the workpiece is machined with the method of ultrasonic vibration machining with variable angle of the disc cutter.

The workpieces obtained under the processing conditions of experimental group 5 and experimental group 6 were photographed using an optical microscope. From Figure 13 and Figure 14, it can be observed that, compared to the workpiece machined without a variable angle in the ultrasonic vibration machining condition, the surface of the honeycomb structure processed without the method of ultrasonic vibration machining with variable angle of disc cutter exhibits tearing defects at the edges and severe burrs. Additionally, the honeycomb cell undergoes deformation. These defects are caused by the large cutting forces generated when machining the inclined surfaces of the honeycomb structure using a disc cutter with a large swing angle. By employing a variable angle of the disc cutter under the ultrasonic vibration machining condition, the forces required for each cutting pass of the disc cutter on the honeycomb structure are reduced, thereby mitigating tearing, burrs, and deformation of the cell.

### 4.3. Quantitative Analysis of Machined Surfaces of NHCs

**Figure 15:**
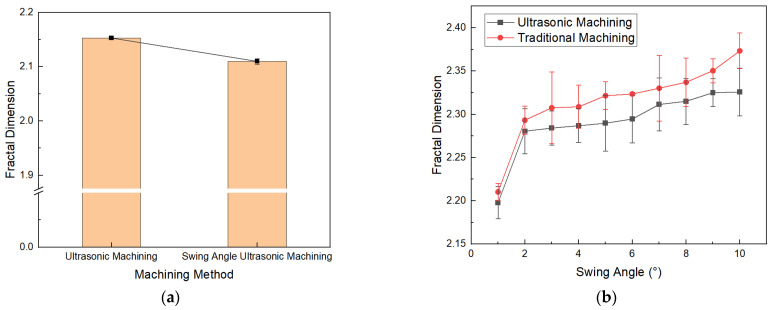
Fractal dimension of the surfaces of NHCs for different machining methods and swing angles. (**a**) Fractal dimension for different machining methods; (**b**) Fractal dimension for different angles.

To quantitatively characterize and compare the surface qualities of NHCs under different processes, the fractal dimension of the machined surfaces was obtained using the box-counting algorithm. Figure 15a shows that the average fractal dimension of the machined surface without the method of ultrasonic vibration machining with a variable angle of the disc cutter is 2.15, while under the proposed machining condition, the average fractal dimension of the machined surface is 2.11. As shown in Figure 15b, the fractal dimension of the honeycomb surface obtained under the ultrasonic machining condition is smaller than that obtained under the non-ultrasonic condition, indicating that ultrasonic machining can effectively improve the surface quality of the honeycomb structure. Additionally, with the increase in angle, the fractal dimension of the honeycomb surface increases. Specifically, under ultrasonic machining conditions, the fractal dimension increases from 2.19 to 2.33, while under non-ultrasonic machining conditions, the fractal dimension increases from 2.21 to 2.37.

Considering that a smaller fractal dimension represents a lower surface complexity and the method of ultrasonic vibration machining with a variable angle of the disc cutter reduces the angle of the tool during cutting, it can be concluded from Figure 15 that ultrasonic vibration machining with a variable angle of the disc cutter can effectively reduce the fractal dimension of the machined surface. Furthermore, when combined with the qualitative characterization of surface quality in Section 4.2, it can be inferred that this machining technique can effectively reduce surface defects and improve surface quality.

## 5. Discussion and Prospect

In this study, a theoretical model was established to qualitatively show that cutting force increases with the increase in angle during the process. Through experimental verification, the consistency between the qualitative theoretical analysis and the experimental results was confirmed. Therefore, the theoretical model established in this study can effectively reflect the reduction in cutting forces achieved by variable angle ultrasonic machining.

However, the theoretical model which was proposed has limitations. In future research, it will be improved to establish a quantitative model that accurately predicts the specific changes in cutting forces with varying angles during ultrasonic vibration machining with a variable angle of the disc cutter. A quantitative model provides many benefits for predicting cutting force and optimizing processing parameters [37,38].

Thus, accurate prediction of cutting forces plays a crucial role in optimizing the variation of angle during the machining process [25], thereby significantly improving the surface qualities of the machined NHCs.

## 6. Conclusions

In this study, the method of ultrasonic vibration machining with a variable angle of the disc cutter was proposed for the milling of Nomex honeycomb composites. The effectiveness of this technique in reducing cutting forces and improving surface quality was demonstrated through the establishment of a theoretical mechanical model and experimental validation. The conclusions are as follows:

(1) The established theoretical model qualitatively analyzed the mechanism of reduction of cutting force, indicating that the method of ultrasonic vibration machining with a variable angle of the down milling disc cutter can effectively reduce cutting forces by using the smaller angle of the tool in each pass compared to directly using a larger fixed tool angle.
(2) The experimental results are consistent with the trend of the theoretical force model. Comparing the process of the fixed 10° angle of ultrasonic vibration machining with the process of a 1° angle in a pass, cutting force can be significantly reduced by 33.5%.
(3) Through qualitative and quantitative analyses of the machined surfaces of Nomex honeycomb composites, it was found that the method of ultrasonic vibration machining with a variable angle of the disc cutter reduces the cutting force in each pass. Thus, it can effectively address surface quality issues encountered during honeycomb milling, such as tearing, burrs, and cell deformation.

## Figures and Tables

**Table 1:** The reduction in the cutting force with decreased angles.

Group	Processing Method	The Reduction of F_z_(%)	The Reduction of F_x_(%)
1	Ultrasonic up milling	33.6	50.3
2	Ultrasonic down milling	33.5	62.0
3	Conventional up milling	26.4	44.1
4	Conventional down milling	27.1	47.0

## Data Availability

The data presented in this study are available from the corresponding authors with a reasonable request.
